# The detection of age groups by dynamic gait outcomes using machine learning approaches

**DOI:** 10.1038/s41598-020-61423-2

**Published:** 2020-03-10

**Authors:** Yuhan Zhou, Robbin Romijnders, Clint Hansen, Jos van Campen, Walter Maetzler, Tibor Hortobágyi, Claudine J. C. Lamoth

**Affiliations:** 1 Center for Human Movement Sciences, University Medical Center Groningen, University of Groningen, Groningen, The Netherlands; 2 Department of Neurology, University Hospital Schleswig-Holstein, Christian-Albrechts-Universität Kiel, Kiel, Germany; 3 Department of Geriatric Medicine, OLVG hospital, Amsterdam, The Netherlands

**Keywords:** Data processing, Machine learning, Motor control

## Abstract

Prevalence of gait impairments increases with age and is associated with mobility decline, fall risk and loss of independence. For geriatric patients, the risk of having gait disorders is even higher. Consequently, gait assessment in the clinics has become increasingly important. The purpose of the present study was to classify healthy young-middle aged, older adults and geriatric patients based on dynamic gait outcomes. Classification performance of three supervised machine learning methods was compared. From trunk 3D-accelerations of 239 subjects obtained during walking, 23 dynamic gait outcomes were calculated. Kernel Principal Component Analysis (KPCA) was applied for dimensionality reduction of the data for Support Vector Machine (SVM) classification. Random Forest (RF) and Artificial Neural Network (ANN) were applied to the 23 gait outcomes without prior data reduction. Classification accuracy of SVM was 89%, RF accuracy was 73%, and ANN accuracy was 90%. Gait outcomes that significantly contributed to classification included: Root Mean Square (Anterior-Posterior, Vertical), Cross Entropy (Medio-Lateral, Vertical), Lyapunov Exponent (Vertical), step regularity (Vertical) and gait speed. ANN is preferable due to the automated data reduction and significant gait outcome identification. For clinicians, these gait outcomes could be used for diagnosing subjects with mobility disabilities, fall risk and to monitor interventions.

## Introduction

Over the last decades, medical and technical developments have extended human lifespan. However, with the increasing number of adults in society, there is a parallel increase in the number of people with serious impairments of mobility, gait, and postural control^1^. Natural aging comes hand in hand with mobility decline and impairments in gait and postural control. When the level of decline in physical and cognitive functions exceeds the degree of decline expected due to the natural aging process, we speak of a geriatric condition. Typical geriatric patients are characterized by co-morbidities such as sarcopenia, cognitive impairment, osteoporosis, weight loss, and frailty^2,3^.

Gait disorders are common in older adults; prevalence increases with age and is associated with increased fall risk, mobility decline, and loss of independence^4^. For geriatric patients, the risk of having gait disorders with an increased fall incidence is even higher^5^. Consequently, objective gait assessment in the clinics has become increasingly important for the diagnosis of motor impairments and the assessment of mobility decline and fall risk^6^, as well as for the monitoring of the efficacy of interventions designed to improve mobility^7^. The most often used gait parameter for disability is gait speed. After age 60, gait speed slows by 16% per decade^8^. In geriatric patients, a gait speed below 1.0 m/s signifies an additional clinical or sub-clinical impairment, such as mobility decline, frailty, recurrent falling, loss of independence and institutionalization^9^. Complementary to gait speed, aging impacts the spatial-temporal characteristics of gait, e.g., walking with a shorter step length, larger step width and increased step time or variability of these parameters^4,10^. However, gait speed may be insensitive and unselective to accurately classify different age and patient groups with specific mobility disabilities.

Advances in technology, in particular with respect to small, light wearable sensors like inertial measurement units (IMU), have considerably aided the practice of clinical gait analysis. Wearable sensors like accelerometer sensors offer new opportunities for clinicians and researchers to record gait over a longer time and allow the application of methods that quantify how gait evolves over time, e.g., the dynamics of gait^5,11^. In addition to gait speed and gait speed related parameters like stride length or stride time, a variety of measures can be derived from these accelerometer signals, that characterize the dynamics of gait through metrics such as, regularity, synchronization, variability, local stability, predictability, smoothness and symmetry^12,13^. These gait outcomes characterize the quality of gait and can be considered complementary to each other. However, not all of these gait outcomes are independent of each other (e.g., gait speed and stride time; regularity and symmetry) and may interact in a non-linear fashion^14^. To analyze multidimensional gait data, specific mathematical approaches are required to define and extract the most informative features of the data and extract parameters that are characteristic for a certain (clinical) population.

The use of machine learning for human gait analysis is nowadays widely explored^15^. Machine learning methods can identify redundancies in a dataset and extract the most informative features of the data by creating new and uncorrelated variables that characterize the original data. Besides, these methods can process high dimensional, non-linear data structures, and based on the learned/trained models, they have the potential to estimate the gait status of new patients^16^. Principal Component Analysis (PCA) has been commonly used to extract significant information from a large number of variables^17,18^. PCA preserves the variability and multivariate features while decreasing dimensionality to make the data analysis more tractable. PCA creates a set of orthogonal bases that capture the directions of maximum variance for the original dataset, and the uncorrected expansion coefficients in the new dataset^18^. However, gait outcomes are not only interrelated with each other but also interact in a complex nonlinear manner^19^. Alternatively, kernel PCA (KPCA) can extract higher-order relations among gait outcomes. The kernel function can employ PCA in high dimensional space but ignores the effect on the non-linear structure^20^. Wu *et al*. showed that KPCA efficiently reduced 23 non-linear gait variables to 17 gait variables, and consequently increased the Support Vector Machine (SVM) classification accuracy from 85% (SVM classification with PCA) to 91%^21^.

Previous studies have also successfully employed machine learning methods to identify gait abnormality in different populations^15^. For instance, Artificial Neural Network (ANN) and SVM are the two most popular machine learning methods in gait analysis^22^. Begg *et al*. applied ANN with linear, polynomial and Radial Basis Function (RBF) kernels to age-classify 30 young and 28 older subjects based on their gait, with a classification accuracy of 75%^23^. In line with this result, SVM classified differences in spatial-temporal, kinematic and kinetic gait variables from 12 young subjects and 12 older subjects due to aging with a 91.7% accuracy^24^. In addition to ANN and SVM, various machine learning methods have been successfully employed for the classification of different patient populations based on gait analysis. K-nearest neighbors (KNN) classification method identified different gait pattern of patients with Cerebral Palsy and Multiple Sclerosis from healthy adults with a classification accuracy of 85%^25^, and of patients with hemiplegia, Parkinson’s disease and back pain with a classification accuracy of 90–98%. However, a limitation of KNN is that it is an instance-based learning method, implying that it only uses the training data for classification but does not learn from it. Similar classification results were obtained from decision tree and Naive Bayes methods^26^. The Random Forest (RF) was used to identify patients with Parkinson’s disease with time-domain and frequency-domain gait features to obtain 98.04% accuracy^27^. In recent studies, several machine learning methods have been employed for classifying fallers and non-fallers with a functional test (such as Timed Up and Go) and questionnaire data to obtained high accuracy of 89.4%^28^.

In sum, these studies support the fact that machine learning methods can be successfully employed for clinical gait analysis to identify differences in gait performance due to pathology, using various types of gait variables. However, in order to be useful for clinical applications, several requirements and constraints need to be considered. In clinical gait analysis, usually the number of variables obtained is high, whereas the number of subjects is relatively low. This may result in an excessively complicated machine learning model with poor predicting performance (overfitting)^22^. With a limited number of subjects, the best choice might be SVM and RF. The effect of a limited number of subjects (data set) is minimized because the classification of SVM depends on the support vectors and the slack variables (not the entire data set) and on the non-linear variables’ distance, to distinguish different groups^29^.

However, the black box problem of SVM implies that before classification, significant features should be detected using, for instance (kernel) PCA^30^. Alternatively, RF can be employed as it is not very sensitive to small data size and is based on decision trees, in which every subject can be repeatedly classified^31^. Nevertheless, RF disregard the intact interactions within and between trees, which might negatively impact the classification performance^32^. Although the black box problem also exists in the hidden layers of ANN^30^, the activation functions such as the tangents hyperbolic can properly analyze the complex interactions among the gait variables to improve the classification performance^33^. A recent study used deep learning to explain gait patterns based on kinematic and kinetic variables.

Due to no recent study investigated aging effect on gait based on dynamic gait outcomes through more quantitative ways, the aims of the present study are two-folded; Firstly, based on an existing dataset 3D-accelerometer signal of healthy young, middle aged older adults and geriatric patients, we evaluated if different groups can be classified based on dynamic gait outcomes. Dynamic gait variables that quantify the quality of gait over time were used as input for the classification of healthy young-middle aged adults, healthy older adults, and geriatric patients. Secondly, we compared the performance of three machine learning models, KPCA in combination with SVM, RF and ANN that can be used for clinical gait analysis.

## Results

### Gait outcome identification and classification with KPCA in combination with SVM

The radial basis function and polynomial function were used in KPCA and SVM, however, no differences were found in KPCA and SVM results between the two kernels functions. In the end, the RBF kernel function was employed in the KPCA and the SVM model.

From the KPCA applied to the original data set of 239 subjects, the first five principal components (PC) captured 97% information of the original 23 gait variables.

**Figure 1:**
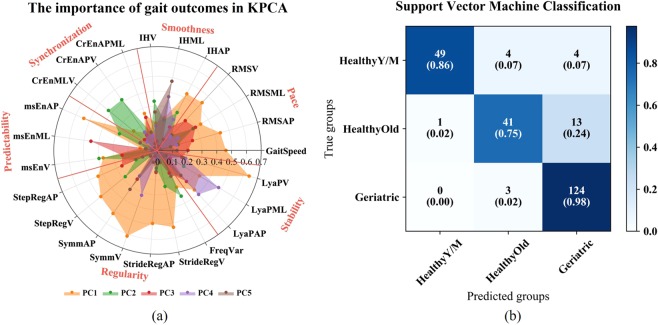
The five colors (**a**) represent gait outcomes contributions to the first five PCs. The orange, green, red, purple and brown areas show the gait parameter distributions on the 5 extracted principal components. The red lines separate these gait outcomes in the field of pace, smoothness, synchronization, predictability, regularity and stability. The abbreviations of the 23 gait outcomes were shown in method, data description. (**b**) shows the classification results for SVM. The blue shading represents the different numbers of subjects from the true groups that were classified into the three age-based predicted groups. The numbers in the parentheses show the percentages of subjects from the true groups that were assigned to the predicted groups.

The different weights of eigenvectors represent the contributions of the gait outcomes on the five PCs (Fig. 1(a)). Gait outcomes achieved weights > = 0.4 were considered significant to the model. PC1 reflected most gait outcomes related to step regularity, step symmetry and amplitude variability (RMS), whereas stability, synchronicity of movement directions, and smoothness were captured by PC2 to PC5.

The extracted PCs of the KPCA were used as the input of the SVM machine learning classifier. To validate the SVM model and decrease the risk of overfitting, LOOCV was used to split the dataset into a training set and a test set for SVM. Figure 1(b) shows the SVM classification (confusion matrix) results for the three groups. The overall classification accuracy is 89.5%. Of the 57 subjects in the healthy young-middle aged group, 4 of them were misclassified and assigned to the healthy older group and 4 were assigned to the geriatric patient group. Of the 55 subjects in the healthy older group, 41 of them were successfully classified into the healthy older group, one was assigned to the young-middle aged group and 13 were assigned to the geriatric group. The 127 geriatric patients were correctly classified with the exception of 3 geriatric patients who were assigned to the healthy older group (Fig. 1(b)).

### Gait outcome identification and classification with random forest

**Figure 2:**
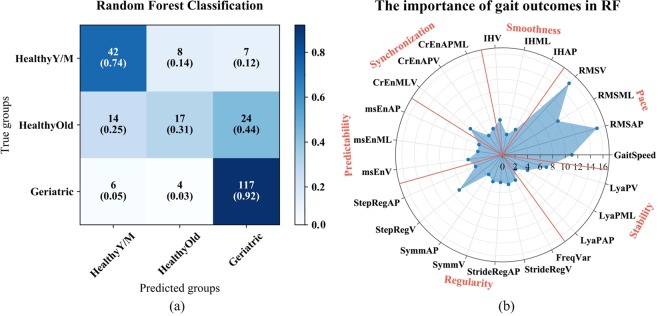
(**a**)shows the classification results of RF with the healthy young-middle aged group, the healthy older group and the geriatric patient group. The blue shading represents the different numbers of subjects from the true groups that were classified to the three age-predicted groups. The numbers in the parentheses show the percentages of subjects from the true groups that were assigned to the predicted groups. (**b**) Value of importance of 23 gait outcomes for RF classification. The axis shows the importance of values. The red lines separate these gait outcomes in the field of pace, smoothness, synchronization, predictability, regularity and stability. The abbreviations of the 23 gait outcomes were shown in method, data description.

Figure 2(a) shows the classification results matrix of the RF method. The RF classification accuracy was 73.6%. Of the 57 subjects in the healthy young-middle aged group, 8 of them were assigned to the healthy older group and 7 were assigned to the geriatric patient group. As is shown in Fig. 2(a), the classification accuracy was worse for the healthy older group. That is 14 healthy older adults were assigned to the young-middle aged group and 24 were assigned to the geriatric group. Finally, 10 of the 117 geriatric patients were misclassified, 6 as healthy young-middle aged and 4 were assigned to the healthy older group.

The gait outcomes that contributed most to the RF classification are presented in Fig. 2(b). Seven of them have larger weights than others (>6), these were the Root Mean Square in AP, ML and V, gait speed, step regularity V, Cross Entropy MLV and Lyapunov exponent V.

### Gait outcome identification and classification with artificial neural network

**Figure 3:**
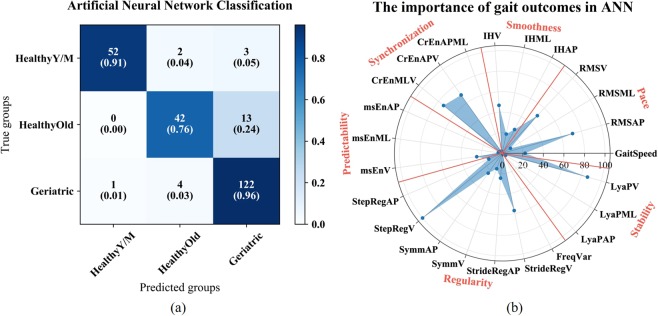
(**a**) Age-classification results for young-middle aged, healthy older and geriatric patients without CI groups in ANN. The blue shading represents the different numbers of subjects from the true groups that were classified into the three predicted groups. The numbers in the parentheses show the percentages of subjects from the true groups that were assigned to the predicted groups. (**b**) Weights of the gait outcomes in ANN classification. The axis shows the important values in ANN. The red lines separate these gait outcomes in the field of pace, smoothness, synchronization, predictability, regularity and stability. The abbreviations of the 23 gait outcomes were shown in method, data description.

The ANN model obtained the best classification performance with one hidden layer, including three units. The overall classification accuracy was 90.4%. Figure 3(a) shows the classification results matrix of the ANN. 2 of 57 healthy young-middle aged subjects were assigned to the healthy older group and 3 were assigned to the geriatric patient group. Similar to the RF, the classification of the healthy older groups was worse, of the 55 subjects 13 were assigned to the geriatric group. Of 127 geriatric patients 122 patients were correctly classified, only one patient was classified as young-middle aged adult and 4 patients were assigned to the healthy older group. According to the ANN classification results, Fig. 3(b) shows 23 gait outcomes in terms of their weight of the ANN classification. The weight of each gait parameter was calculated from the overall layers. In Fig. 3(b), it is shown that 8 gait outcomes contributed much more to the age-based classification than the others. The 8 gait outcomes (weights > 40) were the Root Means Square AP, V, Cross Entropy APV, MLV, step regularity V, Lyapunov exponent V, stride regularity V and The Index of Harmonicity V.

### Evaluation of the machine learning classification approaches

**Figure 4:**
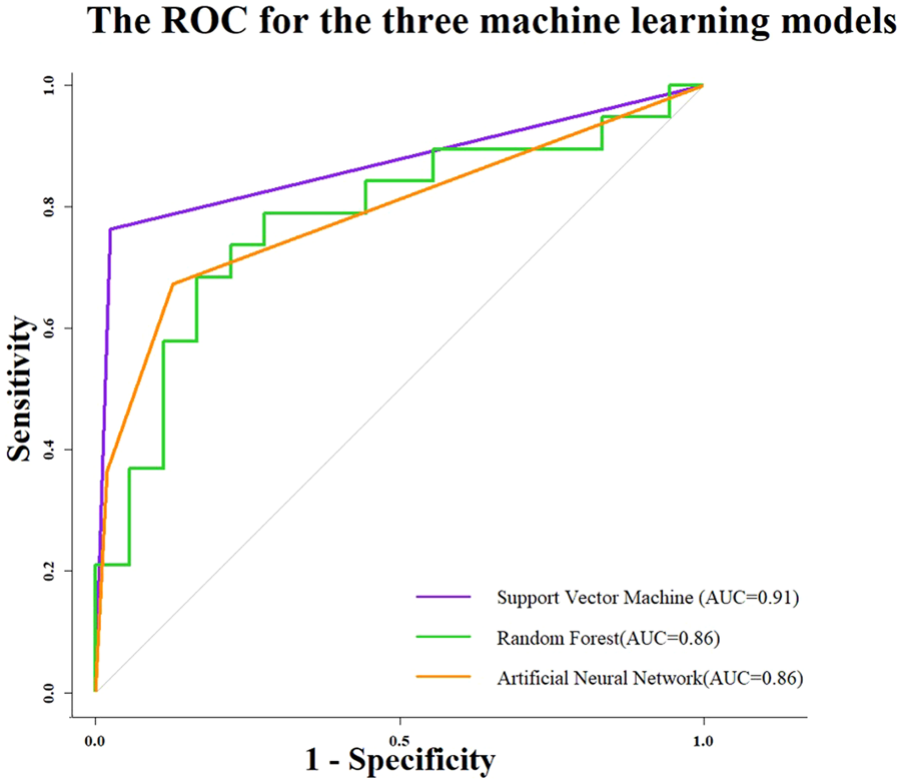
The ROC curves for the machine learning classifier SVM, RF and ANN are shown in the upper panel. The x-axis is the 1-specificity and the y-axis is the sensitivity. The grey dotted line represents the baseline of the ROC curve. Note: the stepwise of ROC of RF is due to the imbalance of sensitivity and specificity and RF classification performance.

The overall classification performances of SVM, RF and ANN were evaluated by the ROC curve. The AUC of SVM, RF and ANN is 0.91, 0.86 and 0.86, respectively. Figure 4 shows the ROC curve for each machine learning classification model.

For SVM, the sensitivity for these three groups is 86% (healthy Y/M), 75% (healthy old), 98% (geriatric) respectively, and the specificity is 99%, 96%, 85%, respectively.

For RF classification, the sensitivity and specificity for the young-middle aged group are 74% and 87% respectively. The sensitivity and specificity for the healthy elderly group are 31% and 93% respectively. The classification from RF in the geriatric patients without CI has the sensitivity and specificity of 92% and 66% respectively.

For the ANN classification, the sensitivity in these three groups is 91%, 76%, 96% respectively, and the specificity is 99%, 91%, 85%, respectively.

### Summary and statistical analysis of the machine learning classification

Table 1 below shows the accuracy and the AUC with a confidence interval (CI) for each model.

**Table 1:** The accuracy and the Area Under the Curve (AUC) with confidence intervals (CI) for each model.

	KPCA + SVM	RF	ANN
Accuracy with CI	89.5% (0.85–0.93)	73.6% (0.62–0.85)	90% (0.82–0.99)
AUC with CI	0.91 (0.81–0.93)	0.86 (0.63–0.83)	0.86 (0.72–0.87)

The abbreviations in Table 1 are: Kernel Principal Component Analysis (KPCA), Support Vector Machine (SVM), Random Forest (RF), Artificial Neural Network (ANN).

Table 2 shows the sensitivity and specificity with a confidence interval (CI) for each model.

**Table 2:** Sensitivity and Specificity with confidence intervals (CI) of each group for each model.

		Healthy Y/M	Healthy Older	Geriatric
KPCA + SVM	Sensitivity with CI	86% (0.15–0.84)	75% (0.56–0.87)	98% (0.70–0.89)
	Specificity with CI	99% (0.38–0.97)	96% (0.68–0.99)	85% (0.05–0.15)
RF	Sensitivity with CI	74% (0.16–0.79)	31% (0.16–0.74)	92% (0.37–0.98)
	Specificity with CI	87% (0.33–0.89)	93% (0.78–0.99)	66% (0.17–0.72)
ANN	Sensitivity with CI	91% (0.05–0.50)	76% (0.39–0.78)	96% (0.52–0.81)
	Specificity with CI	99% (0.17–0.72)	91% (0.44–0.92)	85% (0.07–0.17)

The abbreviations in Table 2 are: Kernel Principal Component Analysis (KPCA), Support Vector Machine (SVM), Random Forest (RF), Artificial Neural Network (ANN), Healthy young-middle age adults (Healthy Y/M).

**Table 3:** The demographics of three age groups.

Class	Healthy Y/M adults	Healthy Old adults	Geriatric without CI
Age range	18–65	>65	>65
Age (mean ± SD)	42.72 ± 16.6	74.58 ± 5.71	79.3 ± 5.81
Number of subjects	57	55	127
Gender	30 M/27 F	25 M/20 F	62 M/65 F

The abbreviations in Table 3 are: standard deviation (SD), male (M), female(F), young-middle aged(Y/M), cognitive impairment (CI).

**Figure 5:**
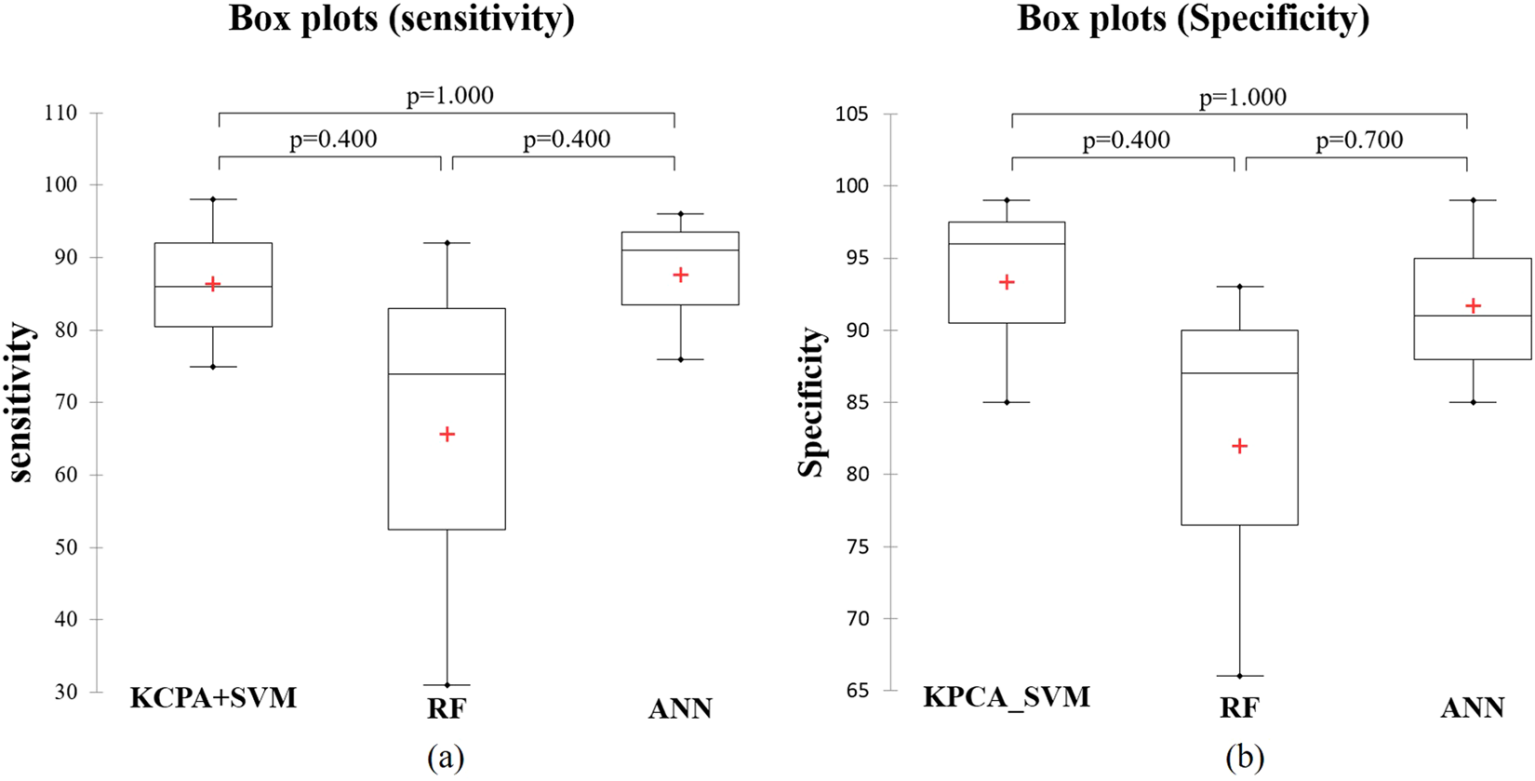
Box plots showing the outcome of the Mann-Whitney U test for comparison of sensitivity (**a**) and specificity (**b**) among groups. The horizontal line is the three machine learning models; the red cross represents means; the central horizontal bars are the medians. The lower and upper limits of the box are the first and third quartiles, respectively, error bars represent the standard upper confidence interval, lower confidence interval in each model. Kernel Principal Component Analysis (KPCA), Support Vector Machine (SVM), Random Forest (RF), Artificial Neural Network (ANN).

Figure 5 shows that there is no significant difference between the three machine learning models’ sensitivity and specificity. This conclusion supports our statement that the classification performance among the three models was similar and valid. However, our statement that ANN has the best classification performance is not supported by this comparison.

Figure 5 (a,b) shows the data with a p-value for the comparison of three machine learning models for sensitivity and specificity, respectively.

## Discussion

The purpose of the study was to identify gait characteristics that could classify young-middle age, older and geriatric adults. To that aim three supervised machine learning approaches were compared in terms of their classification performance, and their ability to identify the gait characteristics of interest.

Overall, KPCA in combination with SVM, RF and ANN, had satisfactory classification accuracy for the three groups (89%, 73.6% and 90%). In addition to accuracy values, AUC values were 0.91, 0.86 and 0.86 for SVM, RF and ANN, respectively.

Based on our results we conclude that machine learning methods (SVM, RF and ANN) when applied to dynamic gait outcomes have the potential to distinguish between groups. The dynamic gait outcomes that were important for classifying the three groups were related to gait synchronization (Cross Entropy between medial-vertical and vertical acceleration), regularity (vertical direction), stability (maximal Lyapunov exponent of the vertical acceleration) and pace (gait speed and the variability of the accelerations (RMS) in anterior-posterior and vertical direction). This result is in line with several previous studies, for instance, healthy older adults and geriatric patients have a gait that is more unstable^34^, more variable and fewer regular^35^ while the slower pace is also the symptoms to distinguish healthy aging and abnormal aging^36^.

The combination of gait characteristics used in the present study had a high specificity ranging from 87% to 99% for healthy adults for SVM, RF and ANN, while the specificity to detect the geriatric patient was lower (66–85%). The sensitivity for healthy older subjects (<76%) for SVM, RF and ANN were worse compared to the patient group (>90%). As shown in Fig. 4(b), the misclassification of healthy older subjects, concerned mostly an assignment to the geriatric patient group and vice versa. The lower sensitivity for the healthy older group could be due to the fact that aging is a continuum, with enormous heterogeneity between subjects in particularly at an older age and in geriatric patients^37^.

The three machine learning approaches showed different classification accuracy, specificity and sensitivity results since they differ in terms of how the algorithms handle non-linearities and interactions between gait outcomes.

Our findings for the KPCA in combination with SVM approach is in line with previous research showing classification accuracy of 91% when comparing young and older adults based on 36 gait spatial-temporal and kinematic gait variables^21^. An advantage of using SVM is that it does not require a large number of subjects (data) because hyperplanes for classification are based on the support vectors and the slack variables^29^. However, all data are configured to a high dimensional space, changing the structure and making it hard to explain which gait outcomes contribute to the classification results^22,30^.

Random Forest (RF) builds multiple decision trees randomly in parallel, considering the correlations of features in every single tree until there is a classification result. Similar to SVM, RF can provide limited datasets because it categorizes the subject by multiple decision trees^31^, the samples in the dataset can be repeatedly selected to be classified in these decision trees^31^. RF is more visible than SVM and ANN, and can output the significant features for the small size of clinical data^32^. The Layer-Wise Relevance Propagation technique in combination with deep learning was used to address the black box problem^38^. However, deep learning usually requires a large amount of data and more suitable for raw data from accelerometer signals. The size of the data and the structure dynamic gait outcomes in this study are not proper for deep learning.

Irrespective of the heterogeneous population, ANN, showed only five misclassifications in the healthy young-middle age group, two were assigned to the healthy older group and three to the patient group showing an impressive performance for this group in particular. In contrast to both SVM and RF, ANN can analyze the complex structure among variables, by using various activation functions (e.g., Tanh, Sigmoid) even though, similar to SVM the variable interactions are not visible^30,33^. Although a large data set is required to find the optimal activation function and avoid overfitting^22^, ANN has the capacity to adapt for the limited dataset with suitable activation functions which may need to be adjusted depending on the type of data^33,39^, to build a small neural network.

While KPCA reduces the data dimensionality as a step toward a subsequent SVM, ANN can automatically reduce the data dimensionality and identify gait outcomes with high weights. RF has good visibility to show significant gait outcomes similar to ANN, however, RF showed lower classification accuracy of healthy older and geriatric patients than ANN. From the results in Fig. 5 we can see there is no significant statistical difference between the three methods. However, ANN has an important advantage, for clinical applications because it does not only have good classification performance, but also those gait features that contributed to the classification can be identified. In contrast, SVM suffers from the “black box problem” which can only be solved by applying the first KPCA. Therefore, we suggest that ANN is more preferable to the current gait data set.

With regard to the selection of machine learning methods, what needs to be taken into account is that the specific selection of machine learning methods depends on the type of data as well as the sample size. If the machine learning method is too simple to model the data, the high bias and low variance model will underfit the classification results. In contrast, if the machine learning method is too complicated for the data structure, the low bias high variance model will overfit the classification results^22^. In clinical data analysis, machine learning methods have the advantage that no prior clinical/gait feature selection is necessary as the features can be automatically selected and used for classification. Yet, to be of clinical relevance, it is important that the results of the machine learning parameters are translated into meaningful clinical knowledge despite the complex interactions among the variables leading to the classification^22,30^.

For the clinician, machine learning methods will not replace but can support and assist human clinical decision-making. For instance, the identified gait outcomes could be used for new patients, for diagnosing fall risk, to monitor the interventions in patients’ daily lives and to optimize the efficacy of specific rehabilitation protocols^22^. For instance, the specific gait outcomes identified by the models can be measured in new patients, to identify the at-risk gait early on, diagnose the potential disorders and to finally determine the patients with a high risk of falling or mobility decline^16^.

To improve the classification of patient groups (e.g., geriatric patients) with numerous co-morbidities in the future, additional variables should be added to the model to improve its clinical value. The three domains, body structure and functions, activity and participation, of the International Classification of Functioning Disability and Health (ICF) provide a framework for including additional variables in the classification model. Future studies should focus on applying pattern recognition methods to identify gait abnormalities in different patient populations based on broader types of parameters reflecting the different domains of the ICF e.g. the Fall Efficacy Scale (FES_I), the Charlson Comorbidity index and further cognition tests^5,40^.

In summary, the present study identified gait characteristics (gait features in synchronization, regularity, stability, variability and pace) that distinguish healthy young-middle aged adults, healthy old adults, and geriatric patients without cognitive impairment using three different classification models. In the future, the gait outcomes identified in this study could be used in the clinics to diagnose patients and monitor interventions in patients. Overall, classifier performance was good, although KPCA in combination with SVM (best AUC) and ANN (best performance) performed slightly better than RF. However, the automated data reduction, classification accuracy and identification of gait outcomes make ANN superior for classifying different age groups based on dynamic gait outcomes. The most difficult group to classify were the healthy older adults, due to the heterogeneity of their gait and the fact that a part of this population might be in a preclinical phase towards geriatric symptoms. Incorporating objective and subjective measures at the different levels of the ICF model in the future, could improve the classification of clinical gait analysis even further, thereby adding to its clinical value.

## Methods

### Data description

Data from different studies^11,41–44^were pooled to create the present accelerometer dataset including 239 participants in three sub-groups: the young-middle aged group (18–65), the healthy older group (>65), and a group of geriatric patients without cognitive impairment (CI) (Table 3). Data from the geriatric patients were obtained between 2009 and 2018^41,42,44^. The young-middle aged and healthy older participants were recruited from a population that didn’t visit the geriatric clinic, by means of advertising in local papers, community centers, and by word of mouth. Data of geriatric patients were obtained from an existing database of a geriatric day clinic in a Hospital. These were patients that visited a geriatric day clinic based on a medical referral by a general practitioner. These patients underwent extensive screening for physical, psychological, and cognitive functions. Criteria for excluding patients from these studies were: (1) inability to walk for three minutes without a walking aid, (2) neurological disorders (e.g., Parkinson’s disease, history of stroke), (3) severe mobility disability caused by pain and/or orthopedic conditions, and (4) the inability to speak and understand the Dutch language. Data of healthy young-middle aged and older adults were obtained from previous studies^11,43^. Procedures followed were in accordance with the Declaration of Helsinki 2000 and all studies were approved by the Medical Ethics Committee of the MC Slotervaart (geriatric patients) or by the ethical committee of the Centre of Human Movement Science Groningen of the University Medical Centre Groningen. All participants of who participated in the studies signed an informed consent form. All subjects followed the same walking test protocols. Subjects walked for three minutes at a comfortable walking speed without aid. During walking trunk accelerations were measured either using the iPod Touch G4 (iOS 6; Apple Inc.), which has a built-in tri-axial acceleration sensor, or using a stand-alone accelerometer unit, the DynaPort hybrid unit (McRoberts BV, The Hague, the Netherlands)^45^.

From trunk acceleration signals, anterior-posterior (AP), medio-lateral (ML) and vertical (V) direction gait outcomes related to the quality of gait, were calculated using custom-made software in MATLAB (version 2014b; The MathWorks Inc.). From the signals, 23 dynamic gait variables were calculated quantifying pace, predictability, regularity, stability, synchronization and smoothness (for a detailed explanation see^11,41^). In brief, gait speed (GaitSpeed) was calculated dividing distance walked (m) by time (s). The Root Mean Square (RMS) is a measure for the variability of the amplitude of accelerations. The Index of Harmonicity (IH) is a measure of gait smoothness. Values range between 0–1, and an IH of 1 reflects a perfectly smooth gait. Multiscale Entropy (msEn) quantifies the predictability at different time scales, testing the complexity of the signal. A value of 0 reflects a completely predictable gait parameter. The Cross-sample Entropy (CrEn) quantifies the degree of synchronization between AP and ML, AP and V, and ML and V accelerations. A value of 0 reflects perfect synchronization between acceleration signals. The maximal Lyapunov exponent (LyaP) represents the local stability of trunk acceleration patterns, as calculated by the Wolff algorithm. Higher values indicate greater sensitivity to local perturbations. The unbiased auto-correlation function of the acceleration signal in AP and V directions was used to calculated gait step or stride regularity (Step/StrideReg) and symmetry (Symm). The signal was phase shifted with a window approximating average step and stride time. Perfectly regular steps or strides are reflected by a value of one. The difference between step and stride regularity showed the gait symmetry, zero representing a perfect symmetric gait. Finally, frequency variability (FreqVar) reflects the relative fluctuations in step frequency. More details of all gait variables with three groups were shown in supplementary information on Table 4. 

#### Gait outcomes standardization

**Figure 6:**
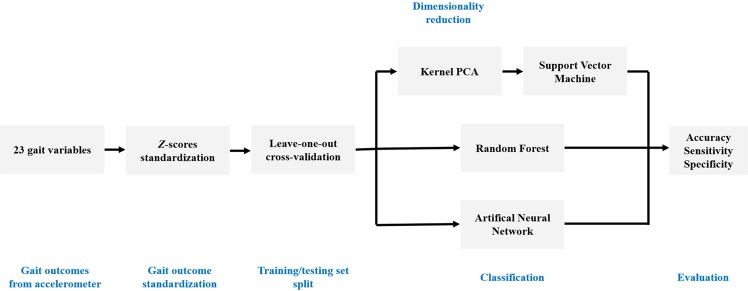
The overall data analysis is illustrated in the flow chart.

Overall, Fig. 6 shows the procedures of the data analysis in the present study. The inter-relationships between the calculated gait outcomes could provide a better understanding of how the process of aging impacts gait. However, before using machine learning approaches to classify age groups based on these gait dynamic outcomes, standardization is needed, since all gait outcomes are calculated at different scales, for example, gait speed in meter/second, IH in scale 0–1. Machine learning algorithms are sensitive to the scales of variables; therefore, the Z-scores method was used to standardize the data.

#### Gait outcomes extraction

To reduce the dimensionality of calculated outcomes while preserving the informative and variability properties and improving classification performance the KPCA was employed. KPCA produces orthogonal bases to capture the directions of maximum variance and the uncorrelated expansion coefficients^21^. Using the KPCA approach, the non-linear input data was mapped to a high dimensional space by different kernel functions, such as linear, polynomial and Gaussian radial basis function (RBF). Then the formal PCA was employed in this new feature space. The formal KPCA algorithm is: the non-linear gait outcomes in the original space are mapped to a high dimensional space Ƒ through kernel functions: ${x}_{i}\to \varnothing ({x}_{i})$ and two inputs ${x}_{i}$ and ${x}_{j}$, which represent two gait outcomes as the examples in original space^46^.

In the present study, two types of kernel functions were taken into account; the first is the polynomial (Poly), *d* is the degree of Poly kernel:

1 $$\begin{array}{c}{\rm{K}}({x}_{i},{x}_{j})={(({x}_{i}\cdot {x}_{j})+1)}^{d}\end{array}$$

The second kernel function is the RBF, $\sigma$ is the width of the RBF:

2 $$\begin{array}{c}{\rm{K}}({x}_{i},\,{x}_{j})=\exp -\frac{{||{x}_{i}-{x}_{j}||}^{2}}{2{\sigma }^{2}}\end{array}$$

The data centered by the following equation:

3 $$\begin{array}{c}\hat{K}({x}_{i},\,{x}_{j})=K-{1}_{N}K+{1}_{N}K{1}_{N}-K{1}_{N}\end{array}$$

The new gait outcomes in the new high dimensional space are:

4 $$\begin{array}{rcl}{y}_{i} & = & \varnothing {({x}_{i})}^{\top }{v}_{j}=\mathop{\sum }\limits_{i=1}^{n}{\alpha }_{ij}\varnothing {(x)}^{\top }\varnothing ({x}_{k})\\  & = & \mathop{\sum }\limits_{i=1}^{n}{\alpha }_{j{\rm{i}}}K(x,{x}_{j}),j=1\ldots d\end{array}$$

In the present study, the entire dataset was used for feature selection because KPCA is an unsupervised process. The components including more than 90% values of the sum of the eigenvalues, were selected as the principal components (PCs). These PCs include almost all information from the original dataset but now with low dimensionality. The distribution of the eigenvectors on each PC shows the contributions of each original gait outcomes to a PC.

#### Cross-validation

The number of subjects in the dataset is relatively low for machine learning approaches. To avoid overfitting^22^, a cross-validation method was used to split the dataset into subsets for training the model, adjusting the model’s parameters and evaluating the classification performance. In this study, the robust cross-validation method “leave one out method” (LOOCV) was applied because it does not randomly partition the data but every subject is used to test the model to reduce the bias. One subset was used to test the performance and *k-1* subsets were used to train the machine learning model. Then for each *k* (equals number of subjects) subset the classification performance was evaluated^25^.

#### Classification

The machine learning methods Support Vector Machine (SVM), Random Forest (RF) and Artificial neural network (ANN) were used in the present study to classify the groups based on gait outcomes. The optimal hyper-parameters are based on the overall classification performance.

#### Support vector machine (SVM)

SVM was used as a classifier to predict subjects’ groups based on their gait performance. In SVM, referring to equation (1) and (2), the two kernel functions map the original data to a high dimensional feature space by finding hyperplanes for different classes, and to maximize the margin between different classes^47^. The output from KPCA was used as the input of SVM classification. The hyperplane for different age scales is defined as:

5 $$\begin{array}{c}\varnothing (x)=sign({\sum }^{}\begin{array}{c}{\beta }_{i}{y}_{i}K({x}_{i},{x}_{j})+b\end{array})\end{array}$$

$K({x}_{i}\,,\,{x}_{j})$ is a kernel function, b is the bias of the training data, β is the coefficient of the separating hyperplane. Then the distance between each class and the hyperplane is maximized:

6 $$\begin{array}{c}\min \,W(\beta )=-\,{\beta }^{{\top }}I+\frac{1}{2}{\beta }^{{\top }}H\beta \end{array}$$

subject to ${\beta }^{{\top }}\,y=0\,$, finally, the new data is classified into a given class:

7 $$\begin{array}{c}H={y}_{i}{y}_{j}K({x}_{i}\,,\,{x}_{j}),i,j=1\ldots M\end{array}$$

All these labeled subjects were classified by SVM with Poly as in (1) and RBF as in (2) kernel functions.

#### Random forest

The Random Forest (RF) method builds various decision trees and merges them to obtain the optimal classification performance. A decision tree suggests the best option to classify the subjects into the three groups. RF combines several decision trees. The subjects were repeatedly classified by each tree. In the end, RF selected the best classification result including the importance of each gait parameter. From the training set ${\{({x}_{i},{y}_{i})\}}_{i=1}^{n}$ (includes n rows ${x}_{i}$ and n columns ${y}_{i}$), a set of m trees were built with individual weight functions ${W}_{j}$ in the individual tree leaf $j$, the prediction $\hat{y}$ of the new testing set $x{\prime}$ is^48^:

8 $$\begin{array}{rcl}\hat{y} & = & \frac{1}{m}\mathop{\sum }\limits_{j=1}^{m}\mathop{\sum }\limits_{i=1}^{n}{W}_{j}({x}_{i},x{\prime} ){y}_{i}\\  & = & \mathop{\sum }\limits_{i=1}^{n}\frac{1}{m}\mathop{\sum }\limits_{j=1}^{m}{W}_{j}({x}_{i},x{\prime} ){y}_{i}{y}_{i}\end{array}$$

The dataset was split into a training and testing set, based on the above described LOOCV method. The original 23 gait outcomes served as M input variables, and m variables (m≪M) were randomly selected to build m decision trees for RF. The value of m remains unchanged during forest growth. Each tree is grown to the most significant extent possible.

#### Artificial neural network (ANN)

The multi-layer perceptron ANN consists of input, output, and one hidden layer with some units. In the ANN system, the artificial neurons are interconnected and communicate with each other. Each connection is weighted by previous learning events and the weight between artificial neurons is adjusted as learning progresses. In the end, the input subjects will be classified into a group through the optimal connection way^49^.

For the components in the ANN, there are neurons, connections, weights, biases, propagation functions, and learning rules. A neuron with label j (here, the label j is healthy young-middle aged, healthy older and geriatric patient without cognitive impairment) receiving an input ${p}_{j}(t)\,\,$consists of the following components: the neuron’s state activation variable ${a}_{j}(t)$, depending on the time parameter t, an activation function $f$ that computes the new activation at a given time t + 1 from ${a}_{j}(t)$, ${\theta }_{j}$ is a fixed threshold, and the net input ${p}_{j}(t)\,\,$giving rise to the relation:

9 $$\begin{array}{c}{a}_{t}(t+1)=f({a}_{j}(t),{p}_{j}(t),{\theta }_{j})\end{array}$$

and then the output is computed by the function:

10 $$\begin{array}{c}{O}_{j}(t)={f}_{out}({a}_{j}(t))\end{array}$$

ANN consists of connections, each connection transferring the output of a neuron i to the input of a neuron j, each connection is assigned a weight ${W}_{ij}$. And a bias ${b}_{0j}\,\,$was added to the total weighted sum of inputs to shift the activation function. To compute the input to the neuron j from the given three age-based groups, the equation shown below adds the bias value:

11 $$\begin{array}{c}{p}_{j}(t)=\sum _{i}{O}_{i}(t){W}_{ij}+{b}_{0j}\end{array}$$

Then the learning process was constructed to modifying the weights and thresholds of the variables within the network.

In the present study, the 23 gait variables were the neurons in the input layer. This model has one hidden layer with three units. There are three output neurons, one for each group, healthy young-middle age adult group, healthy older adult group and geriatric patients’ group. The activation function of ANN is the “Rectified Linear Unit (ReLU).

In this study, ANN classification performance is based on LOOCV, *k-1* sets of training and 1 set of tests. This process was repeated *k* times (*k* = 239). The best hyper-parameters of ANN were decided from the 239 LOOCV iterations of model training and testing, for example, after using LOOCV to train and test ANN, three units in a hidden layer showed the best performance of classification.

#### Evaluation of classification

The accuracy, sensitivity and specificity were calculated to evaluate the performance of the three machine learning classifiers to identify gait for the groups. Sensitivity represents the proportion of those subjects that are assigned to the correct group (true positive rate), and specificity represents those subjects that are rejected to assign to the incorrect group (true negative rate). In the receiver operating characteristic (ROC) curve plot, the y-axis represents the sensitivity and the x-axis represents the 1- specificity. The area under the ROC curve (AUC) provides an overall evaluation of the classification. The baseline of AUC is 0.5, and the perfect machine learning classification model has the AUC = 1.

To statistically test differences between the three machine learning models for sensitivity and specificity we applied a Mann-Whitney U test. The different classes were three models: KPCA in combination with SVM: class 0; RF: class 1; ANN: class 2. Then the three sensitivity values and specificity values across the three groups were regarded as two variables in the model.

## Supplementary information

Supplementary Information.

## Data Availability

The data of all 239 participants and 23 gait variables are not publicly available due to Institute Review Board related matters, but available from the principal investigator Claudine Lamoth (c.j.c.Lamoth@umcg.nl) upon reasonable request.
