# Supplementary material for: The detection of age groups by dynamic gait outcomes using machine learning approaches
Source: Sci Rep. 2020 Mar 10;10:4426. doi: 10.1038/s41598-020-61423-2 (PMC7064519; doi:10.1038/s41598-020-61423-2)
Supplement: Supplementary file 1 — Supplementary Information. [file 41598_2020_61423_MOESM1_ESM.pdf]

The detection of age groups by dynamic gait outcomes using machine learning approaches

Yuhan Zhou[1], Robbin Romijnders[2], Clint Hansen[2], Jos van Campen[3], Walter Maetzler[2], Tibor Hortobagyi[1] & Claudine JC. Lamoth[1]

[1]Center for Human Movement Sciences, University Medical Center Groningen, University of Groningen, The Netherlands. [2]Department of Neurology, University Hospital Schleswig-Holstein, Christian-Albrechts-Universität Kiel, Germany. [3]Department of Geriatric Medicine, OLVG hospital, Amsterdam, The Netherlands. Correspondence and requests for materials should be addressed to Y. Zhou. (e-mail: y.zhou01@umcg.nl).

Supplementary Information

Table 4 shows the mean and the standard deviation of the 23 gait variables are presented for each group.

	Healthy Y/M	Healthy Older	Geriatric
**GaitSpeed**	1.21 ± 0.13	1.13 ± 0.21	0.87 ± 0.23
**RMSAP**	0.18 ± 0.04	0.15 ± 0.04	0.10 ± 0.03
**RMSML**	0.17 ± 0.04	0.12 ± 0.03	0.10 ± 0.03
**RMSV**	0.25 ± 0.07	0.21 ± 0.06	0.14 ± 0.05
**IHAP**	0.84 ± 0.08	0.83 ± 0.08	0.86 ± 0.07
**IHML**	0.66 ± 0.22	0.69 ± 0.20	0.73 ± 0.16
**IHV**	0.73 ± 0.18	0.81 ± 0.13	0.71 ± 0.17
**CrEnAPML**	0.70 ± 0.10	0.65 ± 0.10	0.60 ± 0.11
**CrEnAPV**	0.62 ± 0.10	0.56 ± 0.10	0.54 ± 0.11
**CrEnMLV**	0.65 ± 0.09	0.55 ± 0.09	0.52 ± 0.10
**msEnAP**	1.19 ± 0.17	1.16 ± 0.17	1.26 ± 0.23
**msEnML**	1.91 ± 0.15	1.86 ± 0.18	1.89 ± 0.20
**msEnV**	1.55 ± 0.18	1.41 ± 0.17	1.53 ± 0.24
**StepRegAP**	0.82 ± 0.10	0.78 ± 0.11	0.72 ± 0.13
**StepRegV**	0.80 ± 0.12	0.80 ± 0.14	0.66 ± 0.16
**SymmAP**	3.63 ± 5.59	4.22 ± 4.76	5.87 ± 6.67
**SymmV**	4.21 ± 4.43	4.84 ± 5.78	9.03 ± 8.71
**StrideRegAP**	0.81 ± 0.08	0.76 ± 0.13	0.69 ± 0.14
**StrideRegV**	0.75 ± 0.14	0.75 ± 0.17	0.60 ± 0.18
**FreqVar**	0.04 ± 0.01	0.05 ± 0.02	0.05 ± 0.02
**LyaPAP**	1.77 ± 1.11	1.99 ± 1.19	2.33 ± 1.65
**LyaPML**	2.51 ± 1.44	3.71 ± 1.86	3.66 ± 1.84
**LyaPV**	0.77 ± 0.28	1.11 ± 0.74	1.66 ± 1.07

Table 4. 23 gait features in three age-based groups. The abbreviations in the table 1 are: standard deviation (SD), young-middle aged (Y/M), anterior-posterior (AP), medio-lateral (ML), vertical (V), gait speed (GaitSpeed), the Root Mean Square (RMS), the Index of Harmonicity (IH), multiscale Entropy (msEn), the Cross-sample Entropy (CrEn), gait step or stride regularity (Step/StrideReg), symmetry (Symm), frequency variability (FreqVar), the maximal Lyapunov exponent (LyaP).
